# First report of partial albinism in the blue lobster *Panulirusinflatus* (Bouvier, 1895) from the Mexican Pacific (Crustacea, Decapoda, Palinuridae)

**DOI:** 10.3897/zookeys.784.25082

**Published:** 2018-09-12

**Authors:** Victor Landa-Jaime, Bernabé Aguilar-Palomino, Jesús Emilio Michel-Morfín, Mirella Saucedo Lozano

**Affiliations:** 1 Departamento de Estudios para el Desarrollo Sustentable de Zonas Costeras, Universidad de Guadalajara Gómez Farías 82, San Patricio-Melaque, Jalisco, C.P. 48980 Mexico Universidad de Guadalajara San Patricio-Melaque Mexico

**Keywords:** crustaceans, Eastern Tropical Pacific, Palinuridae, Spiny Lobster, western America

## Abstract

The first case of partial albinism registered in the Mexican Pacific by the blue lobster *Panulirusinflatus* is presented. The specimen was collected on the southern coast of Jalisco know as Punta “El Estrecho”. It constitutes one of the few registered cases of albinism in invertebrates from the Eastern Tropical Pacific.

## Introduction

Albinism is a genetic condition, the result of a mutation on the gene that codifies the enzyme tirosinase, and results in losing the capacity to synthetize melanin, the same pigment that is responsible for the color of skin and hair in animals; therefore the anomaly causes the organisms to adopt a white coloration on the skin and hair and have red eyes (Griffiths et al. 2000). According to the phenotypic characteristic of the individuals, this type of mutation can be expressed in four ways: a) albinism, which is the complete loss of pigmentation throughout the body, b) dilution, where the tonality of the color is reduced as well as other natural pigments, c) squizocroism where the pigment is not expressed but does not affect any other pigments, and d) leucism that is translated in the loss of color of the skin and hair without affecting the soft parts (Buckley 1982). In the latter case, the animals present white skin and hair (Miller 2005, Garcia-Morales et al. 2010). This condition has been recorded in different groups of marine animals like echinoderms (Kehas et al. 2005, Fernández-Rivera et al. 2015), crustaceans (James 2005), fishes (Evangelista-Leal et al. 2013), and sharks (Sancho-Vazquez et al. 2015). Chromatic anomalies have also been registered such as partial albinism, which involve the lack of pigmentation of different body regions (Sancho-Vazquez et al. 2015).

*Panulirus* is a genus that includes representatives of species knows as spiny, rock, or blue lobsters. Most species of the genus have a large worldwide commercial importance (Holthuis 1991, Hendrickx 1995). The blue lobster, *P.inflatus* (Bouvier, 1895), which constitutes the object of this contribution, is frequently observed on rocky reefs with crystalline waters and to 30 m depth (Briones-Fourzan and Lozano-Alvarez 2003). The species is considered to be endemic to the west coast of Mexico, within a specific geographic range from Bahia Magdalena, Baja California, to the Gulf of Tehuantepec, Oaxaca, Mexico (Butler et al. 2011).

## Materials and methods

**Figure 1.**
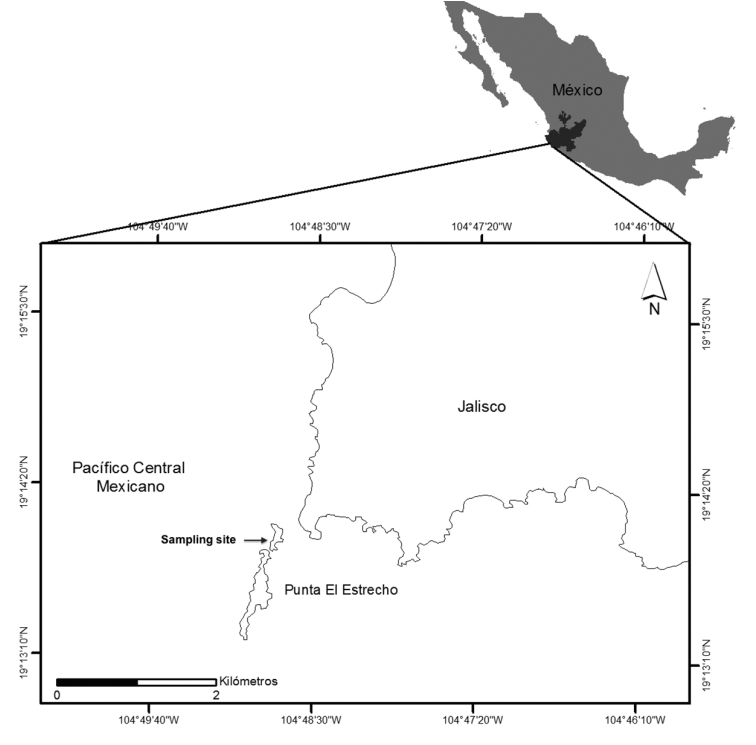
Map of the sampling area, Punta El Estrecho, Jalisco, México.

A specimen of *Panulirusinflatus* was collected from the coastal waters on a site known as Punta El Estrecho, on the southern coast of the state of Jalisco, Mexico (19°06'11"N; 104°29'12"W; Figure 1). The specimen was captured using the hook fishing technique during night submersions at a depth of 15 m on predominantly rocky bottoms using scuba diving on 05 July 2016.

Due to the peculiar characteristics of the specimen, the fisherman who captured it donated the individual to the Universidad de Guadalajara. The specimen was identified using specialized literature (Holthuis 1991, Hendrickx 1995) and preserved in formaldehyde at 10% for a week. The specimen was then washed and transferred to a permanent container in 70% alcohol in the local Invertebrates Collection assigning the following catalogue number: CIDEDSZC1008.

## Results

The specimen examined was a male with a total length of 24 cm and a weight of 300 g. Other measurements taken included: length and width of the cephalothorax 9 cm and 6 cm; length and width of the tail 11 cm and 6 cm; length and width of the claw 4 cm and 3 cm.

**Figure 2.**
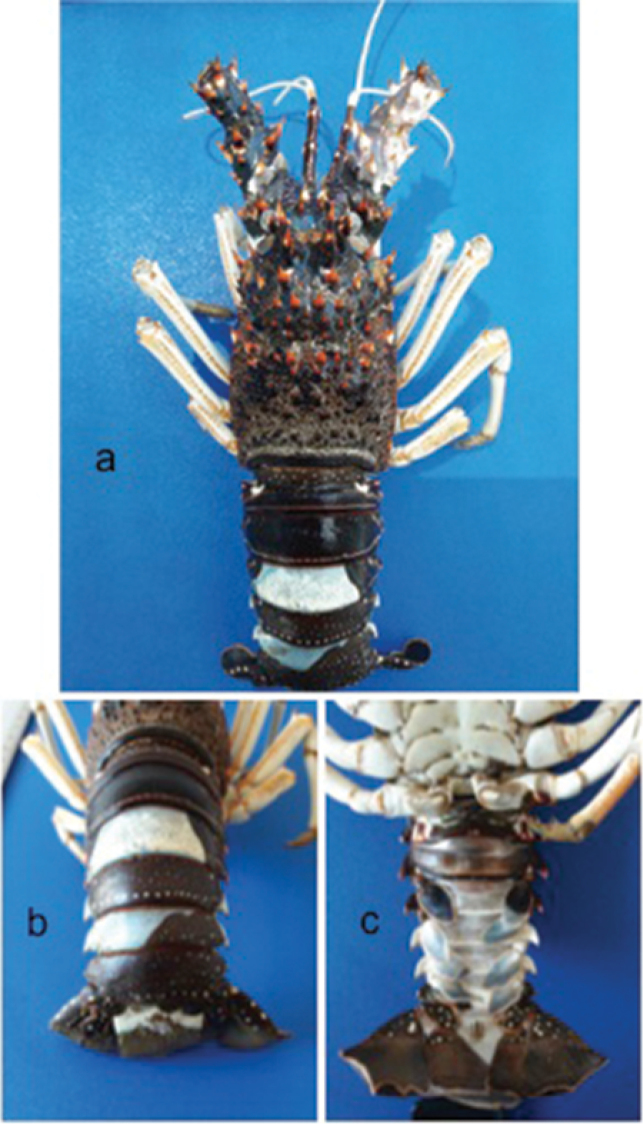
Blue Lobster *Panulirusinflatus*. **a** Complete dorsal view **b** Partial dorsal view **c** Partial ventral view.

Lack of pigmentation was observed on different parts of the body and appendages. Appendages lacking pigments included the flagella of the right and left antennules, the right antenna, the left and right chelipeds, and all pereiopods (Figure 2a). The abdominal somites III and V were partly devoid of pigmentation dorsally and somites III to VI lacked pigmentation ventrally (Figs 2b, c). The cephalothorax lacked pigment only on the ventral part (Figure 2c). The third left pleopod was partially pigmented and pairs of pleopods IV–VI lacked pigmentation (Figure 2c). The telson partially lacked pigmentation on the dorsal and totally on ventral side (Figure 2b, c).

## Discussion

The albinism phenomenon has been reported on vertebrates, and elasmobranches. However, the data are scarce regarding invertebrates (Fernández-Rivera et al. 2015). Isolated cases of albinism on invertebrates have been reported by Kehas et al. (2005) in the land snail *Planorbellatrivolvis*, James (2005) observed this phenomenon in juveniles of crabs *Cancer pagurus*, and Fernández-Rivera et al. (2015) found two specimens with albinism of sea cucumber *Isostichopusfuscus* in the Gulf of California, México. As far as we know, the only reported case of albinism in lobsters refers to *Panulirusjaponicus* (Okamoto and Misyuku 1998).

According to Evangelista-Leal et al. (2013), the occurrence of albinism in fishes can be caused by three factors: random genetic alteration, an effect of marine pollution, or genetic alterations due to the size of the populations. Further studies are required in different species displaying albinism to understand the factors that cause this phenomenon and to evaluate if abnormal pigmentation can be used as an indicator of the quality of the habitat or populations in special situations. In benthic organisms such as species of crustaceans, pigmentation of the body is a vital factor for survival because the organisms use camouflage to avoid predators, when it confused with other animals or their environment (Tan and Richer De Forges 1993).

It must be highlighted that during the previous year in which the specimen was captured an event of the El Niño South Oscillation in the Mexican tropical Pacific occurred (National Oceanic and Atmospheric Administration 2015, Jacox et al. 2016); however, there is no evidence to associate the observed albinism to this oceanographic situation.
